# Open Access Integrated *Thera*peutic and Diag*nostic* Platforms for Personalized Cardiovascular Medicine 

**DOI:** 10.3390/jpm3030203

**Published:** 2013-08-21

**Authors:** Patrick A. Gladding, Andrew Cave, Mehran Zareian, Kevin Smith, Jagir Hussan, Peter Hunter, Folarin Erogbogbo, Zoraida Aguilar, David S. Martin, Eugene Chan, Margie L. Homer, Abhijit V. Shevade, Mohammad Kassemi, James D. Thomas, Todd T. Schlegel

**Affiliations:** 1 Theranostics Laboratory Ltd, North Shore Hospital, Private Bag 93503, Auckland 0622, New Zealand; E-Mails: mehran.zareian@waitematadhb.govt.nz (M.Z.); kevin.smith@waitematadhb.govt.nz (K.S.); 2 Waitemata District Health Board, North Shore Hospital, Private Bag 93503, Auckland 0622, New Zealand; E-Mail: andrew.cave@waitematadhb.govt.nz; 3 Auckland Bioengineering Institute, University of Auckland, Private Bag 92019, Auckland 1142, New Zealand ; E-Mails: r.jagir@auckland.ac.nz (J.H.); p.hunter@auckland.ac.nz (P.H.); 4 Institute for Lasers, Photonics and Biophotonics, 428 Natural Science Complex, University at Buffalo, NY 14260-3000, USA; E-Mail: erogbogbo@gmail.com; 5 Ocean NanoTech, 2143 Worth Lane, Springdale, AR 72764, USA; E-Mail: zapaguilar@yahoo.com; 6 Wyle Science, Technology and Engineering Group, 1200 Hercules, Houston, TX 77058, USA; E-Mail: david.s.martin@nasa.gov; 7 DNA Medicine Institute, 727 Massachusetts Avenue, Cambridge, MA 02139, USA; E-Mail: echan@dnamedinstitute.com; 8 Jet Propulsion Laboratory, California Institute of Technology, 4800 Oak Grove Dr, Pasadena, CA 91109, USA; E-Mails: margie.l.homer@jpl.nasa.gov (M.L.H.); abhijit.v.shevade@jpl.nasa.gov (A.V.S.); 9 NASA Glenn Research Center, 21000 Brookpark Rd, Cleveland, OH 44135, USA; E-Mail: mohammad.kassemi@nasa.gov; 10 National Space Biomedical Research Institute, 6500 Main Street, Suite 910, Houston, TX 77030-1402, USA; E-Mail: thomasj@ccf.org; 11 NASA Johnson Space Center, 2101 NASA Pkwy, Houston, TX 77058, USA; E-Mail: ttschlegel@gmail.com

**Keywords:** pharmacogenomics, echocardiography, electrocardiography, personalized medicine, genomics

## Abstract

It is undeniable that the increasing costs in healthcare are a concern. Although technological advancements have been made in healthcare systems, the return on investment made by governments and payers has been poor. The current model of care is unsustainable and is due for an upgrade. In developed nations, a law of diminishing returns has been noted in population health standards, whilst in the developing world, westernized chronic illnesses, such as diabetes and cardiovascular disease have become emerging problems. The reasons for these trends are complex, multifactorial and not easily reversed. Personalized medicine has the potential to have a significant impact on these issues, but for it to be truly successful, interdisciplinary mass collaboration is required. We propose here a vision for open-access advanced analytics for personalized cardiac diagnostics using imaging, electrocardiography and genomics.

## 1. Introduction

In 2009, the cost of cardiovascular disease in the United States (US) was valued at $475 billion [1]. This high cost is due to modern equipment, diagnostic tests, and therapeutics. Cardiovascular imaging alone accounts for a significant proportion of healthcare expense. In the US, annual imaging costs alone exceed $100 billion [2]. Cardiovascular imaging accounts for 29% of all medical imaging [3] and about one third of all annual medical imaging costs worldwide [4]. In part, these costs are due to proprietary software products, which are a hindrance due to their incompatibility with software from other vendors. Open-source software and digital services have revolutionized many industries, but have not had a significant impact in medicine [5]. Although highly regulated environments are problematic for open-source approaches, open-access, non-proprietary systems would be a significant improvement over the status quo. Lowering cost has also been achieved in other industries through the use of mass customization or personalization. The adoption of personalized services in healthcare has been slower than in other industries in part due to healthcare regulation but also significant barriers alluded to in this paper. 

The process behind personalizing healthcare involves a wide range of technologies. This includes not only genomics but also informatics and imaging. Personalization requires the gathering of high quality, large and accurate datasets, and the use of advanced analytics. As with internet-based technologies, seen in other industries, these can be used to deliver nearest neighbor matching of patient data, using comparisons with similar patients. This is a paradigm shift from the current model of care which uses population values, derived from averaging data. Fueling the transformation to personalized healthcare is a super-convergence of sensor technologies, pervasive connectivity, supercomputing and molecular technologies [6]. This paper discusses emerging open-access technologies which may aid in this transformation. We will elaborate on open access systems for the diagnosis and management of cardiovascular disease, illustrating the use of these technologies with case studies. The analytics systems we outline are open access, vendor neutral, deployable with minimal cost and ideally suited for remote telemedicine.

## 2. Personalized Cardiac Imaging using Ultrasound

Over the last few decades, medical imaging methods have proliferated, with newer technologies offering higher spatial and temporal resolution than previous methods. This has resulted in higher sensitivity than conventional systems but the costs of imaging have soared. In part, this cost has been due to unnecessary imaging and duplication with patients undergoing repeated studies. The availability of medical imaging is also often restricted to secondary or tertiary care hospitals. Advanced and prolonged medical training is often required to interpret imaging studies, which leads to high running costs. Unfortunately, efficiency and cost reduction has not been a significant focus for industries producing imaging systems. Until recently, few imaging systems were portable or deployable outside of specialized hospitals, however that is rapidly changing. 

**Figure 1:**
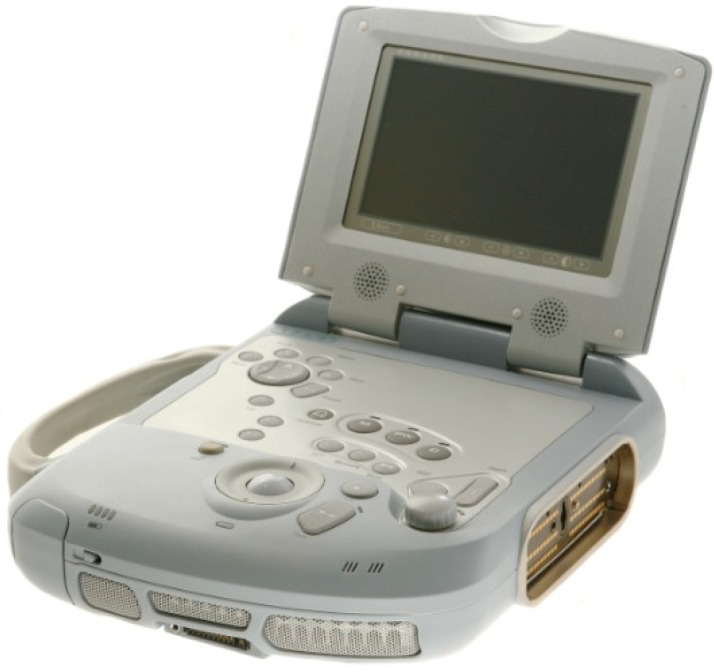
Portable Imaging Systems. ZONE Sonography Technology acquires ultrasound data up to 10 times faster than conventional systems and implements data acquisition and management in software rather than hardware. The limiting factor for image and analytical processing therefore becomes computer processing unit (CPU) processor speed, which increases at an exponential rate (Moore’s law).

Of all the available imaging technologies such as computed tomography, ultrasound, nuclear medicine, and magnetic resonance imaging, ultrasound is the most cost efficient and sustainable [7,8]. Ultrasound imaging of the heart, known as echocardiography, has been a core imaging modality in cardiology for over 50 years. Despite the advent of other diagnostic imaging methods, ultrasound has remained an essential tool, due to its versatility and ability to assess both structure and function of the heart in real-time. Technological advances in computer processing units (CPUs) and miniaturization have led to the development of ultraportable ultrasound systems which are capable of examining not only static organs, but also the heart (Figure 1). Advances in data acquisition and processing have allowed high frame rate imaging of the heart, which has enabled highly accurate assessment of cardiac function. 

**Figure 2:**
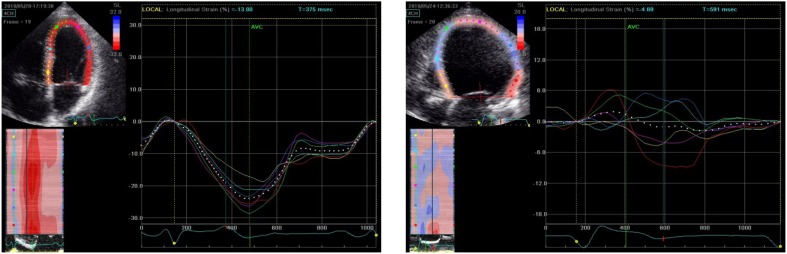
Regional and Global Longitudinal Strain. Examples of instantaneous regional longitudinal strain (color coded segmental lines) and global longitudinal strain (white dotted lines) measured over a cardiac cycle (EchoPAC™, General Electric). The heart (left) demonstrates a normal shape, volume and maximal global longitudinal strain with segmental homogeneity. The heart (right) demonstrates a severe cardiomyopathy with spherical remodeling, increased ventricular cavity volume, reduced maximal global longitudinal strain and segmental heterogeneity.

Echocardiography is used for the diagnosis of structural heart disease, coronary artery disease, valvular pathology and abnormalities of cardiac function. The assessment of cardiac function, using left ventricular blood ejection fraction, is one of the most commonly used measures in clinical medicine. However, early changes of cardiac function cannot be observed using standard methods of assessment such as ejection fraction. These subtle changes can be detected using advanced measures of cardiac function such as ventricular wall deformation, otherwise known as strain [9]. Strain is measured using methods of ventricular wall tracking on 2D or 3D moving images of the heart and calculates a percentage difference between a baseline length and instantaneous length of a segment of the heart (Figure 2). Strain-based assessment of cardiac function has been used in research studies as a method to predict the presence of coronary artery disease [10], and early cardiotoxicity in the use of anthracycline and HER2neu targeted biological chemotherapies (e.g., Trastuzumab) [11,12]. This can also be considered personalized since patient-specific changes in strain values are more predictive of the presence of disease than comparisons with population values. Hence, chemotherapy related cardiotoxicity can be inferred when strain deviates from individualized baseline values. This is only possible due to the high degree of accuracy and reproducibility of strain measurements [13]. Accurate assessment of cardiac function is also an important clinical variable factored into many clinical algorithms, for example decisions related to surgery, implanting defibrillating pacemakers, and drug therapy. In addition, cardiac function has an emerging role in pharmacogenomic algorithms [14].
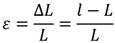

where *ε* is *normal strain*, *L* is the original length of the fiber and *l* is the final length of the fiber. Measures of strain are often expressed in a negative percentage of total change in length of a cardiac segment or of all segments (global strain).

As mentioned, cardiac strain has shown promising clinical utility in several settings; however, a major challenge that hampers widespread utilization is the availability of manufacturer-specific software with various tracking algorithms. In “A Suggested Roadmap for Cardiovascular Ultrasound Research for the Future”, one proposed technique to overcome vendor-dependency is to perform strain assessment on images in the widely utilized Digital Imaging and Communications in Medicine (DICOM) format [15]. DICOM images are derived from the native polar scan-line (raw) data and contain data in Cartesian coordinates. As part of this effort, our team is developing an open-access software system to assess subtle changes in cardiac function in astronauts on the International Space Station (ISS) [16]. The group effort is part of NASA’s Integrated Cardiovascular project.

## 3. The Integrated Cardiovascular (ICV) Project

The impact of long-term microgravity on cardiovascular function may become a critical limitation to human space exploration. Ultrasound is well suited for space exploration due to its portability and versatility in imaging multiple organs quickly. For this reason, ultrasound imaging platforms have been installed aboard the International Space Station [17,18]. Our own work involves the use of these platforms within the Integrated Cardiovascular (ICV) Project and the larger framework known as the Digital Astronaut program. The Digital Astronaut program aims to generate computational models of various organ systems, by using a modular XML (extensible markup language) file format. The process of analyzing the human heart in space involves a number of steps. First, there is astronaut training, currently consisting of five sessions wherein crewmembers focus on gaining familiarity with the study protocol and remote guidance procedures. Second, real-time guidance of in-flight acquisitions is provided by a sonographer in the Telescience Center of Mission Control. During this step, physician investigators with remote access are also able to relay comments on image optimization to the sonographer. Live video feed is also relayed from the ISS to the ground via the Tracking and Data Relay Satellite System, with a 2 s. transmission delay. The expert sonographer uses these images along with two-way audio to provide instructions and feedback. Third, images are stored in non-compressed DICOM format (750 MB per study) for asynchronous relay to the ground for subsequent off-line analysis [19]. Offline analysis involves the measurement of a number of parameters including strain. This analysis has shown there is a reversible, time-dependent reduction in global longitudinal strain in astronauts spending several months in space [20]. 

**Figure 3:**
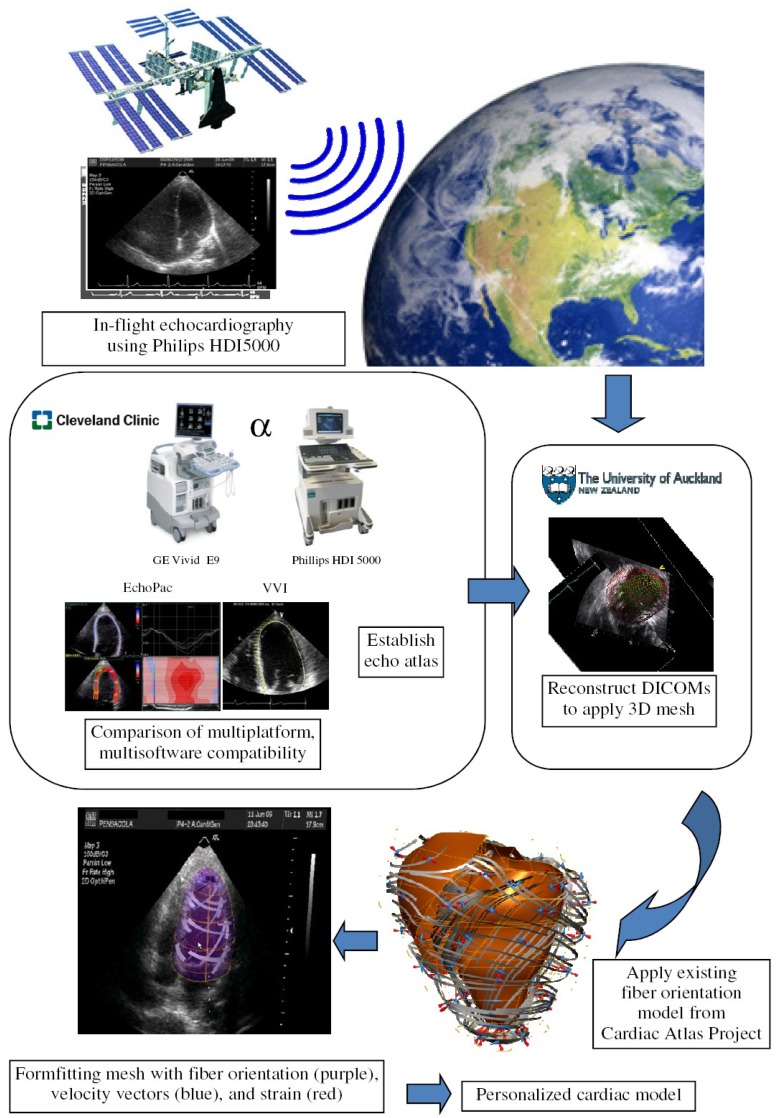
Workflow for Gathering Echocardiography Data from the International Space Station (movie) and Integration into the Strain Based Model (movie).

Further offline analytics are performed through the application of a personalized cardiac model to the echocardiographic images and the use of an electronic echocardiographic atlas, to compare each individual model with an existing patient database (Figure 3). Superimposing a model of cardiac structure and function onto echocardiographic images is advantageous for a number of reasons. Cardiac models have been used to improve edge detection algorithms by interpolating and extrapolating myocardial borders between imaging frames, and models have improved volumetric assessment. In addition, modeling the material properties of the heart tissue can be used to estimate strain. Models are also valuable as multiple data inputs can be superimposed onto models and extrapolated to make predictions based on prior observations. 

The echocardiographic software we are developing aims to be low cost, open access and widely available, utilizing to the greatest extent possible an open source approach. The expectation is that this software, developed for space-based applications, will ultimately have use in ground-based clinical practice as either a third party plugin, within echocardiography laboratories, or as a plugin for a standalone product, within a Cloud-based environment. 

Cloud-based echocardiography reporting (e.g., StudyCast^TM^) can have a transformative impact on the delivery of healthcare in the third world. For example, the ASE-REWARD (American Society of Echocardiography) program was a brief intervention which involved screening patients in a remote rural community in India, using pocket or portable cardiac ultrasound [21]. Over two days during this project, 1,030 echocardiograms were performed and interpreted by remote readers in different time zones, using a Cloud-based server. The advantages of using the Cloud-based server included flexible storage and remote viewing of studies. Although not used in this project, Cloud-based technology also has the potential to apply advanced analytics, which often requires high performance computing and is readily available in the Cloud. The logistics of remote outreach telemedicine in rural communities has many of the same issues as monitoring astronaut health in space and the parallels between these two projects have been highlighted.

## 4. The Human Physiome Project

The cardiac modeling detailed here is also part of a broader international collaboration, known as the International Union of Physiology (IUPS) Human Physiome Project [22]. The IUPS Physiome Project is a worldwide effort to define the physiome through the development of databases and models that facilitate the understanding of the function of genes, cells, organs, and organisms as an integrated whole. The project is focused on compiling and providing a central repository of databases, linking experimental information and computational models from many laboratories into a single, self-consistent framework. The long term aim of this project is to develop modules for creating a Virtual Physiological Human for applications in personalized medicine. 

By extracting metadata from echocardiographic DICOM files, including cardiac chamber dimensions, and applying strain analysis to left ventricular mechanics, data rich models of the heart can be created. Preliminary steps have been achieved in the creation of these models, using data gathered from the ISS. These have demonstrated close agreement with clinically validated strain software systems [23]. Integrating data from electrocardiography and genomic sources is the next intention for this project.

## 5. Advanced Electrocardiography

Electrocardiography is an important diagnostic tool often used early in the assessment of cardiac disease. Although conventional resting electrocardiography (ECG) has an important role in managing acute coronary syndromes and chest pain, it has well-recognized limitations in the detection of heart disease [24]. For both isolated and pooled ECG abnormalities, the sensitivity of conventional resting ECG as a predictor for coronary artery disease (CAD) and left ventricular hypertrophy (LVH) has been too low for it to be practical as a screening tool [25,26]. Furthermore, while normal conventional resting ECG findings have excellent negative predictive value (NPV) for left ventricular systolic dysfunction (LVSD), the simultaneously poor positive predictive value (PPV) of abnormal conventional ECG findings also limits conventional ECG’s utility in heart failure screening [27,28]. 

Over the past decade, researchers working in conjunction with NASA have implemented a comprehensive suite of advanced ECG (A-ECG) diagnostic software techniques on a single software platform [29]. Currently, all of the incorporated techniques can be performed simultaneously and their most important results are intelligently integrated in real time by using software-based statistical pattern recognition procedures [30]. When applied clinically, A-ECG takes advantage of pre-existing databases of conventional ECG, derived vectorcardiographic and several other advanced ECG results from thousands of patients with known, imaging-proven cardiac diseases and from thousands of healthy subjects. By referencing any “new” patient’s results to results already in the aforementioned large databases, and through use of state-of-the-art signal and statistical processing, A-ECG techniques have become diagnostically more powerful than conventional ECG alone [29,31]. A-ECG results can also be derived from digital ECGs collected on most conventional ECG equipment already in the installed base, and from digital ECGs currently stored only as “conventional ECGs” within electronic health records. A-ECG also shares the same advantages that have made conventional ECGs ubiquitous: *i.e.*, noninvasiveness, inexpensiveness, diagnostic capabilities that are multifunctional, and amenability not only to telemedicine but also to patient-centered and patient-driven healthcare. 

**Figure 4:**
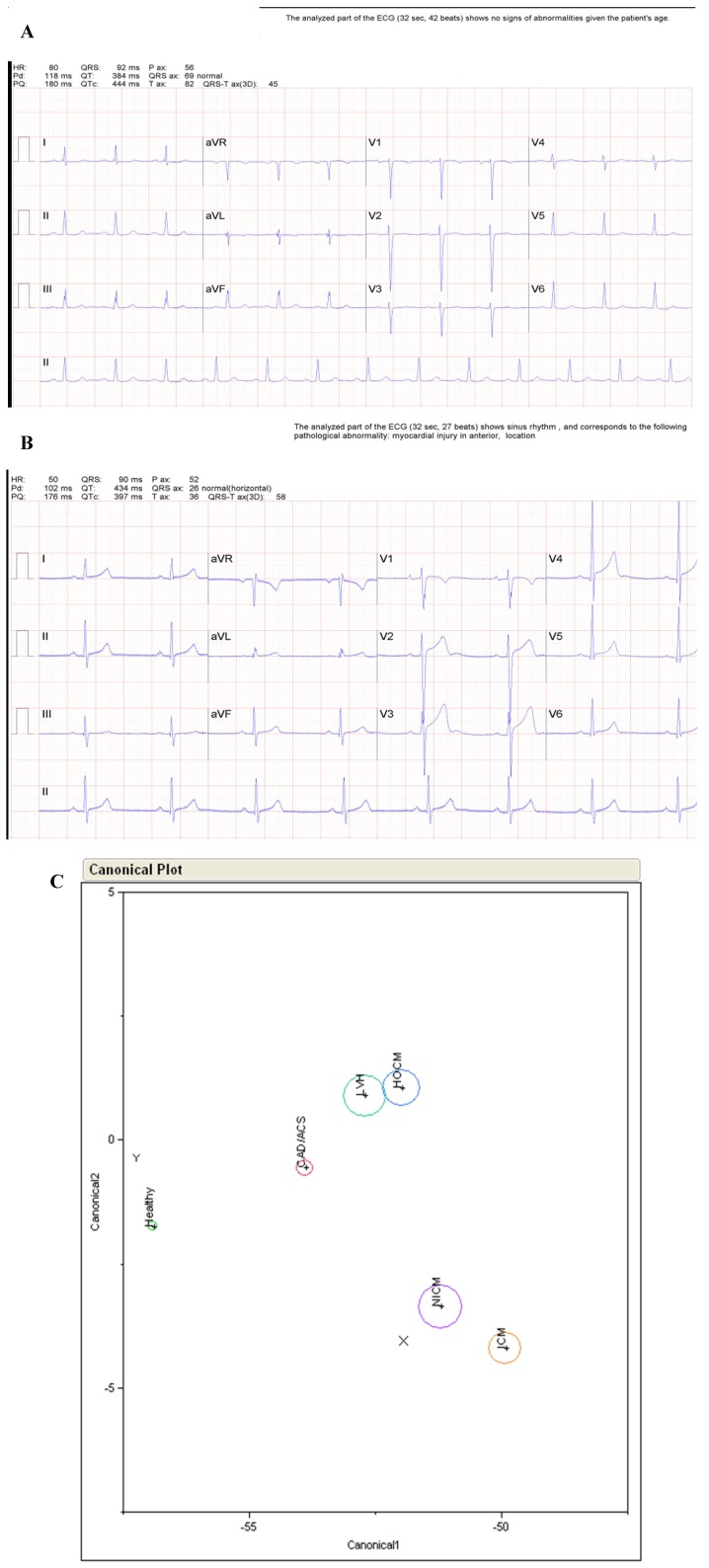
Additional Diagnostic Clarity Provided by Advanced ECG. (**A**) Conventional 12-lead ECG from a 57-year old male with imaging-proven non-ischemic cardiomyopathy and low left ventricular ejection fraction (25%). The conventional ECG software’s automated interpretation, (see top of **A**) is false negative in this case, even though poor R-wave progression is present; (**B**) Conventional 12-lead ECG from a 30-year old male triathlete, with a normal cardiac MRI. The conventional ECG software’s automated interpretation (“myocardial injury in anterior location”) is false positive in this case because the ST segment elevation present in some precordial leads is due to early repolarization, not “myocardial injury”; (**C**) Advanced-ECG-related linear discriminant analysis (DA) results from the same ECGs shown in (**A**) and (**B**).

Figure 4 shows two example case studies illustrating the additional diagnostic clarity that A-ECG can provide over strictly conventional ECG. The first case study (A) in Figure 4 shows an appropriate reversal by A-ECG of a “false negative” automated diagnostic call on the part of the conventional ECG, while the second (B) shows an appropriate reversal by A-ECG of a “false positive” automated diagnostic call on the part of the conventional ECG. In (C) the traditional linear DA (“Canonical Plot”) depicted takes into consideration results from only certain key parameters available from the 10-s “snapshot” ECG (such as the derived VCG-related spatial QRS-T angle and other “3D ECG” parameters), and it specifically identifies non-ischemic cardiomyopathy (see X marker on Plot) as the likely underlying condition for the 57-year old patient, whose conventional ECG is shown in (A). Similarly, for the 30-year old patient whose conventional ECG is shown in (B), the A-ECG DA result correctly predicts the presence of “health”, not disease (see Y marker on Plot). All DA-related results were also confirmed by corresponding results from statistically more robust A-ECG-related logistic regressions [29].

The circles in the canonical plot diagram represent DA centroid results for various different populations in a large underlying database: Small green circle = Healthy population; red circle = Coronary Artery Disease (CAD) and/or Acute Coronary Syndrome (ACS) populations; aqua circle = Left Ventricular Hypertrophy(LVH) population; blue circle = Hypertrophic Cardiomyopathy (HOCM) population; purple and orange circles = Non-Ischemic (NICM) and Ischemic (ICM) Cardiomyopathy populations, respectively.

## 6. Cardiovascular Biomarkers for Personalized Medicine

A biomarker is defined as a measurable characteristic which reflects the physiological status of an individual. Although medical imaging is considered a biomarker, the term is more commonly used to denote blood-based clinical chemistry. In 1902, Archibald Garrod, one of the founders of clinical chemistry, introduced the concept of personal “chemical individuality”, and foresaw a future where genetics and chemical variability would become intertwined [32]. Conceptually, this was thinking far ahead of its time and now could be taken a step further with the inclusion of medical imaging. Whilst medical imaging assesses anatomic structure and function, blood-based biomarkers, such as nucleic acids, proteins or metabolites, are also capable of inferring the same, without imaging the whole organ or organism. There is an emerging realization that integrated imaging and clinical chemistry has significantly greater value than contemplating these fields as separate.

Although medical imaging is a commonly used diagnostic tool, there is a well-recognized limitation to its capabilities. Pathological change, visible at an organ level, is generally an example of late-stage disease processes. For example, the imaging modalities used in the diagnosis of coronary artery disease, such as stress echocardiography and nuclear cardiography, are used to assess advanced disease and are incapable of detecting subtle early coronary artery disease or cardiac metabolism. Despite significant efforts to increase spatial and temporal resolution to improve sensitivity, returns have diminished for additional expense on medical imaging. A sustainable, low-cost option may however be to integrate low-cost imaging systems and biomarkers together, into diagnostic algorithms [33]. An excellent example of a biomarker which provides additive value to diagnostic imaging is the cardiac-specific troponin assay. In addition to aiding the diagnosis of myocardial infarction, troponin plays a role in the diagnosis of incipient heart disease; for example, anthracycline induced cardiomyopathy [11,12]. An emerging evidence base suggests that troponin and imaging, such as nuclear scintigraphy [34] and strain-based echocardiography, may be complementary in detecting this cardiotoxicity [12]. Although appealing, the integration of multimodality imaging with biomarkers in clinical practice is inhibited by a lack of point-of-care instruments and evidence-based diagnostic algorithms. 

Similar to the limitations of diagnostic imaging, technological advances in laboratory systems are also reaching a roadblock. Improvements in technology have increased the sensitivity and precision of analytical systems, and resulted in earlier medical diagnosis; however, conversely, this has been at the cost of reduced specificity, and a reduction in diagnostic accuracy for single conditions. Single analyte biomarkers—for example, prostate specific antigen—have been used widely for prostate cancer screening. However, due to a high false positivity rate and potential for treatment harm, there is a general trend away from using this assay for population screening [35]. Troponin assays, used in the diagnosis of myocardial infarction (MI) and cardiovascular disease, again provides a prototypic example of this problem [36]. Although more sensitive and specific than previous biomarkers (CK, CK-MB and AST), the first generation Troponin assay lacked dynamic range at low concentrations. Early generation assays were therefore only able to confirm a diagnosis, some hours after an event. Newer generation troponin assays have greater sensitivity and analytical accuracy for measuring lower quantities of troponin [ng/L for high sensitivity (hs) assays and pg/L for ultrasensitive assays] [34]. Although the increased sensitivity of assays has resulted in earlier diagnoses, the tradeoff has been reduced specificity, with positive results occurring in noncardiac conditions such as sepsis [37]. Therefore, the use of hs-troponin has led to inappropriate diagnoses, investigations and treatments, which heighten the risk for iatrogenic injury. Next generation, ultrasensitive assays, are likely to further exacerbate this problem. The solution to this conundrum is likely to come from the emerging fields of metabolomics and proteomics. These technologies appear promising in making both early and accurate diagnoses with high levels of sensitivity and specificity [38,39,40,41]. Metabolomics for instance has been used in an experimental setting for the early diagnosis of myocardial infarction, chronic stable coronary artery disease and metabolic wellness [39,40,42].

Metabolomic and proteomic profiling involves the broad quantitation and identification of thousands of metabolites and proteins. This is often performed using mass spectrometry or nuclear magnetic resonance, both of which are capable of identifying thousands of analytes rapidly and inexpensively. Metabolomic profiling, using mass spectrometry, is already in clinical use and has revolutionized microbial identification in many clinical laboratories [43]. Labor intensive and slow historical methods of identifying microbes can now be performed within seconds using mass spectrometry, with spectral profiles matched to a database of known microorganism metabolite profiles. Making sense of the high volume data resulting from experimental metabolomic and proteomic studies is however complex and requires advanced bioinformatics. For biomarker discovery, these pattern recognition algorithms often require high performance supercomputing which itself is becoming more readily available outside of high-end research laboratories.

## 7. Bioinformatics and Supercomputing

As already discussed, single biomarker assays are commonly used in the diagnosis of complex disease. However, due to the limitations described, this approach is recognized as reductionistic. This becomes even more apparent when complex and unpredictable biology is taken into account. Current practice is to compare quantitative values of individual biomarkers with reference ranges, or thresholds, to make a diagnosis. These thresholds are often based on knowledge of variability within healthy and diseased populations. Very rarely, individualized biomarker values are used, which are based on prior baseline testing of an individual patient, age-specific reference ranges, or known variability within race or gender. The use of multiple (multiplexed) biomarkers, to make a single diagnosis, is even less common, though there are some examples where this is used. A complete blood count can be considered as a multiplexed biomarker test and patterns within a complete blood count are used to diagnose a wide range of hematological illnesses. These gross patterns are discernible by experienced clinicians and hematologists; however, the complete blood count is also a useful example to demonstrate the value of advanced pattern recognition. Complete blood count patterns, discovered using supercomputing and advanced biostatistics, have been shown to be highly predictive of cardiovascular outcomes [44]. Although single parameters—such as red cell distribution width—within a complete blood count are known to have modest predictive value [45], patterns otherwise imperceptible to humans appear to have a significantly higher predictive capacity for cardiovascular risk. 

Similarly, larger datasets, such as those seen in genomics, proteomics and metabolomics have the potential to reclassify and restratify patients with disease [46]. Unfortunately, the high volume of data from proteomic and metabolomic analysis rapidly exceeds the capacity of the human brain to comprehend and although the principles for using these technologies have been around for many years, the ability to rapidly visualize and interpret this data has hindered its emergence in the clinical setting. However, as already discussed, metabolomic profiling has found a place in the clinical laboratory in the identification of bacterial microbes. This clinical profiling requires only a desktop computer to run, however for biomarker discovery and the handling of very large datasets, such as whole genome sequences, high performance computing is required. In recent years, access to supercomputing resources has become more widespread and costs have dropped significantly. Web-based Cloud services such as Amazon Web Services, Microsoft Windows Azure and Google compute provide low cost storage and supercomputing to those with expertise and internet access. 

**Figure 5:**
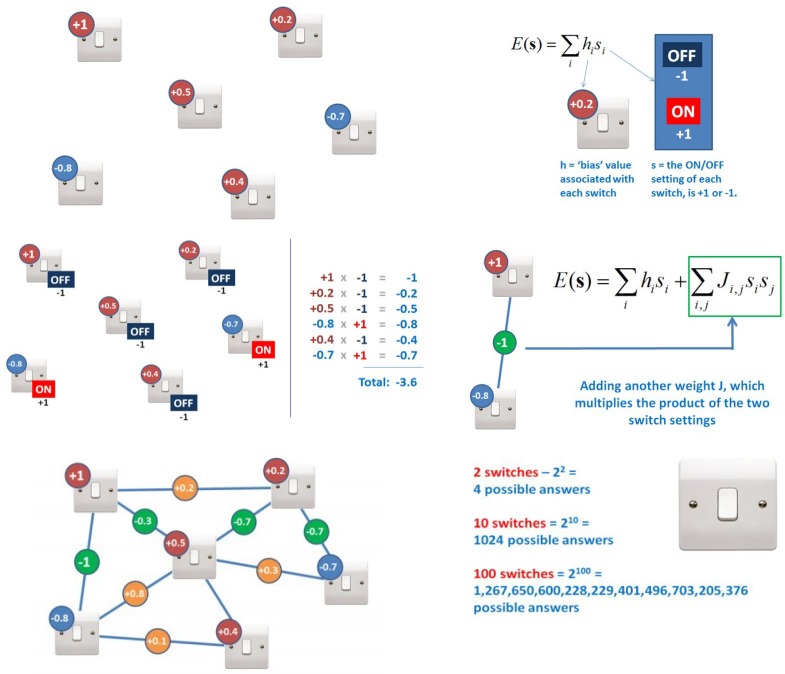
Optimization Algorithms, Interactions and Exponential Computations. An optimization problem is posed when a number of variables, e.g., SNPs, in aggregate account for an outcome such as a disease or drug response. An example of such variables shown below demonstrates this in the context of binary light switches, each with an independent weighted influence (top left). The sum of their influence on the outcome is calculated using the equation (top right) and summed in a linear fashion (middle left), in a relatively simple calculation. In the discovery phase, these weightings would not be known and would be discovered iteratively, with high computational needs. If, however, the factors interact in a network (middle right) a far higher number of permutations exists. The number of permutations is an exponential function of the number of variables and requires exponential computations (bottom left and right) for each added variable. A number of potential solutions exist for a given minimal dataset, within which the optimization algorithm attempts to find the best solution. Quantum computers, such as D-Wave systems [48], are ideally suited for the exponential computations required for optimization algorithms and are anticipated to be revolutionary for medical diagnostics and imaging [49]. Graphic reproduced with permission from D-Wave systems, “Hacking the Multiverse”.

Furthermore, emerging next generation computer systems, such as Quantum computers, are also expected to be widely available through remote access. Quantum computation may revolutionize the approach to complex optimization problems commonly encountered in the search for genetic epistasis [47] and gene-environmental interactions (Figure 5). 

## 8. Cardiovascular Genomics and Handheld Point-of-Care Platforms

Apart from inherited heart diseases, such as the Long QT syndrome, hypertrophic cardiomyopathy and familial cardiomyopathy, genomic medicine has not featured prominently in general cardiology practice. Pharmacogenomics for commonly used medication promises the greatest potential for introducing genomics into the mainstream. This has an emerging role in patients taking several common cardiovascular medications, including clopidogrel [50], statins [51], dabigatran [52] and warfarin [53]. The last two of these drugs are frequently used in patients with atrial fibrillation, which in itself is rapidly being rewritten as a genomic condition. Common variants in chromosomes, 4q25, 1q21 and 16q22, have all been associated with atrial fibrillation in genome-wide association studies (GWAS) [54]. As an example, carriers of multiple variants within all of these loci are at five to six-fold increased risk of atrial fibrillation [55]. Clinical factors, combined with these common variants, are an important component of clinical risk prediction models. These clinical factors include patient height, left ventricular function (assessed by echocardiography) and left ventricular hypertrophy (assessed by either echocardiography or electrocardiography) is often available electronically and is well placed to be accessed by decision support software [56]. In addition to predicting disease, 4q25 variants have also been associated with the response to specific antiarrhythmic drug treatments [57], efficacy of direct current cardioversion (DCCV) and response to pulmonary vein isolation [58,59,60]. In the future, it is likely that genetic data will be integrated with imaging metadata to enhance clinical decisions regarding invasive procedures [56,58].

**Figure 6:**
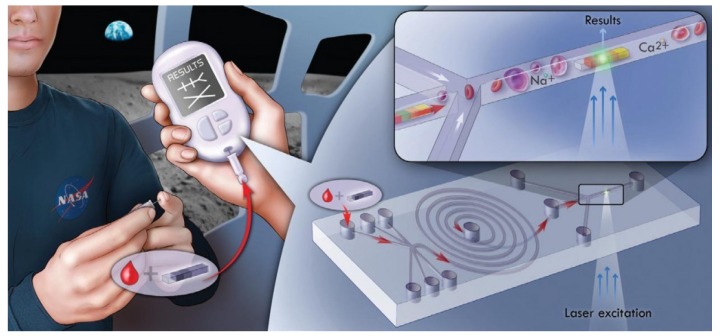
Point of Care Diagnostic Platform. The rHEALTH sensor is a self-contained, general-purpose device utilizing microfluidics and nanotechnology. It delivers multianalyte single molecule detection capabilities and provides demonstrated 100-fold better limits of detection (LOD) than current ELISA and bead-based immunoassay technology, with 896-fold multiplexing.

Integration of multiple diagnostic methods, such as imaging and ‘omics, appears to be the key to the advancement of sustainable medicine. However, the cost of purchasing multiple instruments with separate functions limits the potential for applying this at the bedside or in the clinic. There are few laboratory platforms that can perform metabolomic, proteomic and genomic analyses in a single instrument. At present, none perform these simultaneously; although promising, handheld point-of-care technologies are emerging which may radically change that status quo. The rHealth sensor is an example of such a technology, which is currently in preclinical development. The instrument utilizes a novel microfluidics platform, nanotechnology and low cost sensors to provide rapid, sensitive and accurate analysis of multiple biomarkers (Figure 6). A convergence of mass spectrometry-type analytics and nanotechnology may be the key to reaching the breadth of analyte detection, high sensitivity and specificity required for applications in the field of personalized medicine [61,62]. Since mass spectrometry has yet to become a handheld instrument, it is probable that portable integrated systems, including multiple highly sensitive sensors, will perform this role.

**Figure 7:**
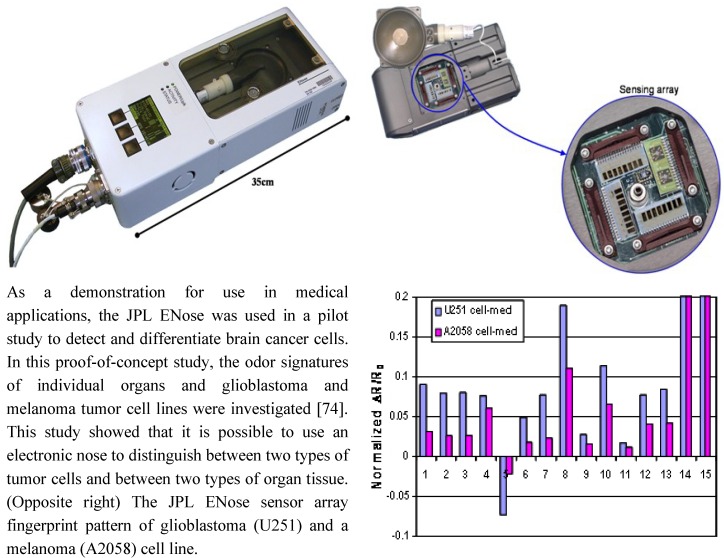
Chemoresistive Sensor Array. The JPL Electronic Nose (ENose: below left) [71,72] is an event monitor designed and built for near real time air quality monitoring in crew habitats aboard the space shuttle/space station. This is an array-based sensing system which is portable (low volume and power), and designed to run continuously and autonomously. The sensor array is optimized to monitor (detect, identify, and quantify) for the presence of selected chemical species in the air at parts-per-million (ppm) to parts-per-billion (ppb) concentration ranges. For each sensor (below right), resistance is recorded, the change in resistance is computed against a background, and the distributed response pattern (fingerprint or smellprint) of the sensor array is used to identify gases and mixtures of gases [71,72,73].

Biological fluids and volatiles, other than blood, are already used in many clinical assays. Exhaled breath analysis is considered an undervalued source for biomarkers related to health. Existing indications for exhaled breath testing include nitric oxide detection in chronic asthma and hydrogen testing in patients with gastrointestinal bacterial overgrowth [63,64]. Recent research has shown that exhaled breath shows high promise as a biomarker for other health states [65]. Breath metabolite profiles, or fingerprints are extremely individualized, showing the possibility for the distinction of disease and tracking an individual’s health over time [66]. There are many potential applications for the use of breath analysis in the management of cardiovascular disease. For instance, selected ion flow tube mass spectrometry (SIFT-MS) exhaled breath analysis has recently been shown to have diagnostic utility in patients with heart failure [67]. Breath markers of oxidative stress may have diagnostic use in patients with acute coronary syndrome [68] and can demonstrate the benefits of traditional relaxation therapies and beneficial changes in gene expression [69]. Portable electronic nose technology, such as the Jet Propulsion Laboratory (JPL) ENose, has the potential to translate these findings into a low cost, home-based diagnostic or therapeutic monitoring test (Figure 7). Self-monitoring one’s health at home, with constant biofeedback, would be the ultimate vision for the future of healthcare, however significant impediments must be overcome before such breath testing can become a standard of care. These include methodological issues such as collection standards, and instrument sensitivity [70]. In addition, the absence of a roadmap or atlas of breath gases and metabolites in health and disease reduces the clinical applicability of these emerging technologies. 

## 9. Network Medicine: A Holographic Universe

Quantifying nucleic acids, proteins and metabolites for diagnostic purposes is only one aspect of the use of genomic technologies. A further step is to take the dynamic information, delivered from genomic analysis, and visualize the complex interactive networks that make up biological systems, otherwise known as systems biology. This ‘systems’ method has shown its utility in defining the pathophysiology of early disease, by demonstrating intermediate pathophenotypes (precursors) that eventuate as clinical disease. Network medicine is the title given to this field of study and its breadth covers not only molecular networks, but also at a larger scale, social networks. The impact of combining these approaches can be profound. For example, mycobacterial gene sequencing and social network analysis has been shown to be useful in identifying super-spreaders of multidrug resistant tuberculosis [75]. Conceptually, interactive networks are a foreign concept to clinicians. These networks however demonstrate some consistent features such as being scale-free, clustered and also demonstrate emergent behavior, which is otherwise not evident from studying isolated network components [76]. A network analysis of the spread of obesity [77] and smoking behavior [78] has shown the ‘contagiousness’ of an individual’s actions amongst their nearest neighbors and demonstrates the potential for targeted interventions which could have wider community impact. 

Integrating multiple sources of genomic data has proven to be difficult, but network models have the capability of visualizing the highly complex and expansive universe of human physiology. This has provided tantalizing new perspectives on chronic disease diagnosis and treatment. Integrating multiple ‘omic datasets into network models has been illuminating, but clinical publications using integrated ‘omic data are rare. For example, Lin *et al*. used genomics, proteomics and metabolomics to identify the pathways affected by end stage cardiomyopathy [79]. Whilst this study failed in its primary goal to demonstrate a difference between end-stages of ischemic and nonischemic cardiomyopathy, its approach was remarkable in showing that biological pathways can be inferred from whole blood samples, rather than from specific organ tissue. Similarly, gene expression signatures from whole blood have been shown to reflect identical genes expressed in diseased aortic atheromatous tissue [80]. This ‘functional holography’ of organ systems, through blood sampling, could be a significant step forwards in reducing the invasiveness and expense of medical diagnostic testing. The ability to infer end organ disease using gene expression profiling from blood is already currently available for the diagnosis of coronary artery disease [81] and cardiac transplant rejection in patients [82]. 

Pharmacology can also be considered from a systems biology approach. Systems pharmacology would view the action of a drug to be multifaceted, with drug action occurring amongst a network of dynamic genes. High throughput pharmaceutical screening with genomics has created databases through which a drug’s gene expression profiles can be matched with a corresponding ‘opposite’ disease profile. Rather than using the empiric approach of evidence-based medicine, these profiles can be used to rapidly identify new disease indications for ‘old’ medication. This process otherwise known as computational drug repositioning has been used to identify novel drug indications, which have shown efficacy in animal models and could have future clinical applications [83,84]. 

**Figure 8:**
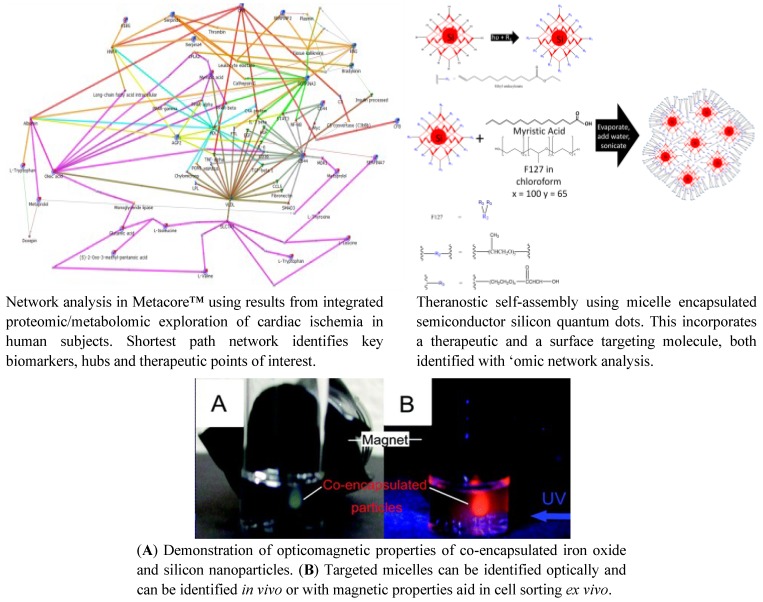
Rational Design of a Theranostic using Proteomic and Metabolomic Network Data.

In other ways, genomics has revealed the molecular underpinnings to disease and has led to the development of gene-specific therapies e.g., Kalydeco, Vertex Pharmaceuticals for cystic fibrosis. Unfortunately, for therapies such as Kalydeco, this drug development has proven to be prohibitively expensive [85]. Although this maintains the perpetuation of medicine for the wealthy, network medicine and computational drug repositioning have greater potential. Finding new uses for generic medication, through an inductive rather than deductive reasoning, may deliver cost efficient personalized medicine. This comes at an opportune time as the current paradigm of treating populations, on a one-size-fits all basis, appears to be failing. Off-target effects of drugs, such as statins, are becoming evident in population studies e.g., causing myopathy, renal failure [86] and diabetes [87]. Targeting network modules, rather than organs or diseases per se, with next generation therapeutics may lead to a significant advance, compared with the contemporary methods of treatment which have multiple unexpected effects. Nanomedicine holds an additional promise in that regard as it can be applied in not only diagnostic but also therapeutic applications. As the field of diagnostic and therapeutic medicine continues to converge the potential of theranostic (*thera*peutic diag*nostic*s) nanomedicine is being realized and a number of these agents have already reached regulatory approval in the field of oncology. Eventually, traditional high throughput screening of drug compounds may be surpassed by a more rational approach to therapeutic drug design which may occur using integrated genomic analyses on an individual patients (Figure 8) [88]. Computationally repurposed drugs may potentially be generated on site and delivered in a highly targeted basis, reducing overall exposure to agents, which may be systemically toxic [89]. Although this will take some years to emerge in clinical practice, these fields of basic science, once thought to be science fiction, seem on track to becoming a reality.

## 10. Case Presentations

Five cases are presented to demonstrate the diagnostic potential of the advanced diagnostic systems described. Case 1 shows the value of VECG analysis in the early sensitive detection of acute myocardial infarction in a case which otherwise would have been considered borderline for the diagnosis of STEMI by ECG and biomarker criteria. Case 2 describes a case where a diagnostic assumption was made based on a patient’s exposure to a cardiotoxic chemotherapy agent. More expensive methods of diagnosis such as MRI may have been averted with the use of A-ECG and remission with treatment and recrudescence could have been tracked over time. Case 3 demonstrates a patient with a borderline diagnosis, which was unclear using a coarse, contemporary method for assessing left ventricular function. Integration of imaging and gene sequencing data in this case may have had a higher diagnostic yield. Case 4 describes a patient presenting with the complication of stent thrombosis, following coronary artery stenting. This adverse event was, in part, due to drug failure caused by the patient carrying a loss of function enzyme responsible for metabolising a drug into its active form. A subsequent switch was made to an alternative treatment, not as dependent on the same enzyme. Ideally matching the right drug to the right patient would have occurred prior to this adverse event occurring. Case 5 describes a young patient with a rare genetic cardiomyopathy and muscular dystrophy. Her treatment has included contemporary heart failure medication, though these fail to address the underlying cause of her illness, a Lamin A missense mutation. The expression of this nonessential gene product could potentially be targeted with network medicine and computational drug repositioning. Alternatively targeted gene therapy could be delivered to knock down gene function. Unfortunately, as this patient suffers from a rare, orphan disease, a large scale, randomized controlled trial is unlikely to be performed. Alternatively, an n = 1 trial [90] with close scrutiny of efficacy and toxicity could be applied, but this is an anathema to the current evidence-based model of care.

## 11. Discussion

With aging populations, constrained financial systems, and enormous wastage within the current medical model of care, secondary care is becoming increasingly unaffordable and unsustainable. Greater decentralization of healthcare resources and supported autonomy will be necessary to reduce this burden cost. This will only be possible through advances in portable diagnostic systems, which are becoming increasingly accurate and smart due to advances in nanotechnology, electronics, bioinformatics and computer processing. Crowdsourced solutions such as the Tricorder X prize will likely propel this super-convergence of imaging, genomics, and low cost sensors into home-based integrated diagnostic systems [91]. In this paper, we have outlined a number of future space medicine technologies and demonstrated the value of integrating imaging, informatics, electrophysiology, and molecular technologies in a diagnostic to therapeutic continuum. Portability, low-cost, remote guidance with the preservation of autonomy are common needs in the delivery of community healthcare of both westernized and developing countries. These systems could potentially see wide usage in both ends of this spectrum. In addition, the systems demonstrated here are open-access and capable of receiving inputs from multiple vendor sources. 

Through the use of case studies, we have shown the technologies described in this paper to have higher diagnostic accuracy than current methods. Supportive clinical data of these and other similar technologies is soon to emerge on a population basis. The novelty of these technologies and potential for disruption to established clinical practice however may impede their introduction in healthcare systems, where revenue is generated from high revenue investigations. For example a truly patient-centered approach to outpatient cardiovascular diagnostics in the future might consist of resting A-ECG together blood based biomarkers testing; followed up if a problem is identified with personalized ultrasound-based cardiac imaging. This non-invasive and inexpensive approach maintains sufficient accuracy whilst avoiding exposure of patients to any radiation during nominal, non-emergent outpatient diagnostic workups. It thereby also fulfills the crucial Hippocratic concept of “first do no harm”. While concerns regarding cost have frequently been raised, abetted by the generally helpful maxim that “less is often more”, it should be understood that when the results of these inexpensive technologies are intelligently integrated by clinicians who comprehend the “less is more” message, they typically result in prompter diagnosis, less need for invasive and expensive follow-up imaging. In addition, this leads to the improved stratification of patients into responder *versus* nonresponder groups for avoidance of expensive but potentially unnecessary and harmful treatment options. 

Academic clinical medicine has become incredibly resistant to change, in part due to years of advocating approaches that are at best “evidence-based” only in the sense of populations, not in the more important sense of the individual. For instance, there is now an expectation in cardiovascular medicine that clinical adoption cannot occur until a large, multicentre, randomized-controlled outcomes trial has been performed. In addition, individual hypotheses are tested sequentially, a model that is ultimately not feasible for personalized medicine. Adoption of many of these technologies may initially need to occur more on the basis of inductive and personal rather than deductive and population-based approaches, realising that with close scrutiny and experience, the ideal applications, niches and algorithms for the use of these powerful technologies will reveal themselves over time. 

Even though the platforms for genomic science and bioinformatics are maturing, there are many areas unprepared for its implementation. Education of medical students, physicians in training and specialists is essential to ensure that the benefits and utility of these fields are not lost. Decision support and result visualization also need to be seamless and understandable. Novel engaging methods of reporting, using mobile devices, and interactive and intuitive visualizations will be needed. Augmented and virtual reality holds significant potential in this regard as an aid for teaching purposes, not only in remote and automated guidance, but also in demonstrating visuospatial problems such as 3D cardiac anatomy and physiology. For example, virtual and augmented reality have been used in sonographer training for echocardiography and will probably be the only way for skilled procedures to be delivered into the hands of the unskilled [92].

The common theme within these technologies is personalization. Although it is widely held that this concept relates only to genomic medicine, it encompasses a far greater whole which includes delivering personally relevant information to patients. Reducing a person to images, physiologic data and DNA has added immense complexity to managing patients in practice and has the potential to dehumanise the patient interaction as less time may be spent in face to face contact [93]. Simplifying the delivery of genomic information by masking the underlying systems and reducing the perceived complexity will be required to make these technologies work in a busy clinical environment while maintaining a personal touch. 

**Figure 9:**
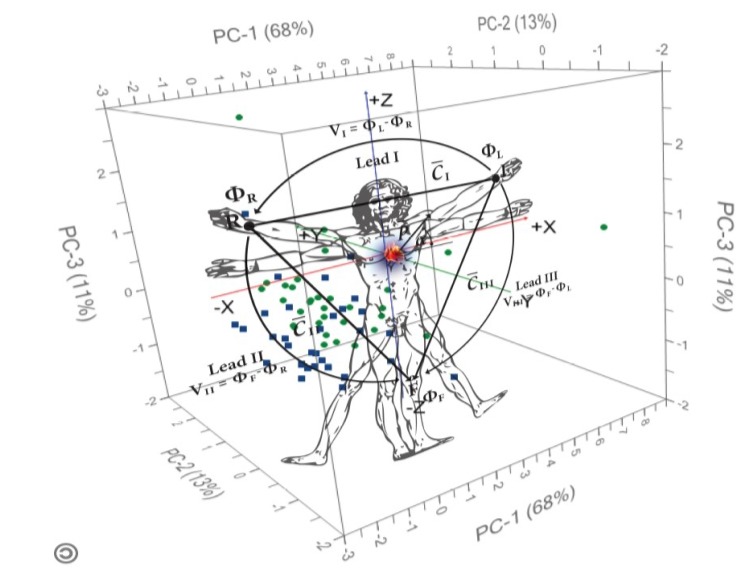
The Modern Vitruvian Man. Da Vinci’s Vitruvian man, often used as a symbol of humanity, was drawn to demonstrate the proportions of the human body but with the purpose of relating man to nature. In his anatomical drawings and in Vitruvian Man, he considered a cosmografia del minor mondo (Cosmography of the microcosm). With increasing evidence that the human body comprises not only its own vast number of nucleic acids, proteins, metabolites, *etc*., but also those of its microbiome [95], Da Vinci’s perception of the human body as a universe of interacting molecules is at once both late-medieval and ‘modern’. It would seem that the only universe capable of containing the human person is an irreversibly personalizing universe [96].

In general, entrenched dogma changes slowly; however, excessively reductionist and population-centric approaches to healthcare are increasingly giving way to newer, more integrative and individualized approaches. Whilst previously lacking an evidence base, some traditional forms of healthcare have long argued for the same focus on the individual as a collective whole. Remarkably, this has many parallels with genomics and perhaps, unsurprisingly, these traditional forms of healthcare are being shown to have a genomic basis [94]. Whilst westernized medicine has discarded centuries-old perceptions of health as excessively simple, these older, more integrative, ‘systems’ perceptions may in fact have significantly greater relevance to the future of medicine than realized (Figure 9). 

## Case 1: Acute Myocardial Infarction

**Figure 10:**
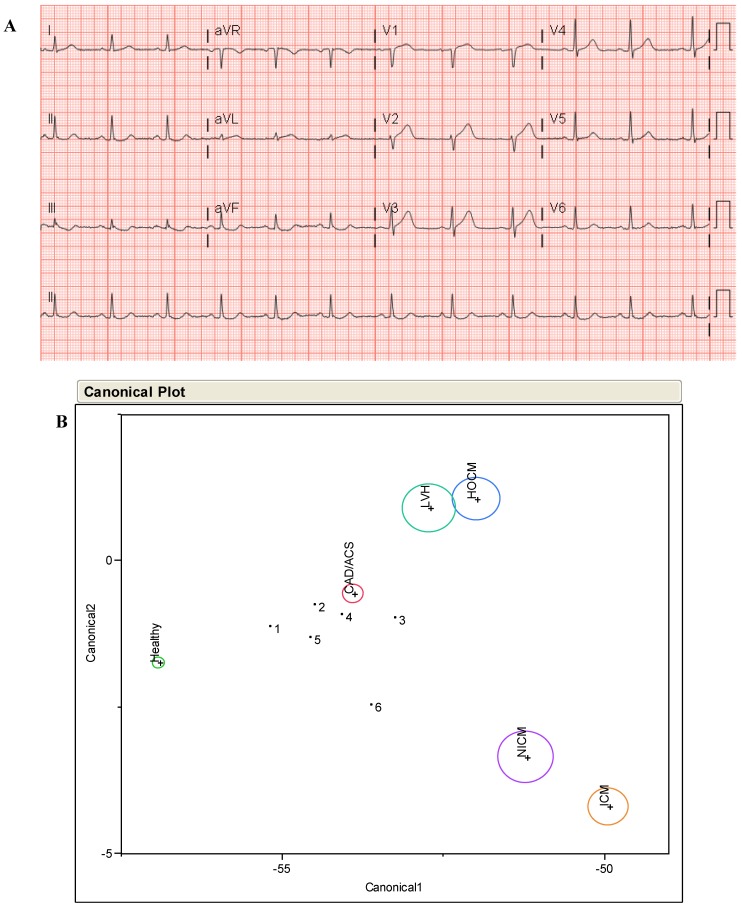
Conventional ECG for Case 1 (**A**); (**B**) A-ECG-related discriminant analysis results for six serial ECGs in Case 1 using multiple advanced ECG parameters. Red circle = Coronary Artery Disease and/or Acute Coronary Syndrome (CAD/ACS) population; Aqua circle = Left Ventricular Hypertrophy (LVH) population; Blue circle = Hypertrophic Cardiomyopathy (HOCM) population; Purple and Orange circles = Non-Ischemic (NICM) and Ischemic (ICM) Cardiomyopathy populations.

A 57 year old man with no previous cardiac history presented to hospital with 47 min of severe, central chest pain. A conventional ECG performed at admission (Figure 10A) had a negative automated call for myocardial infarction (MI) but showed isolated 1 mm ST elevation in leads V1 and V2. An hs-troponin I (hs-TnI) measured 42 ng/L (reference range 0–40). A provisional diagnosis of acute MI was made and medical treatment given. However, neither primary PCI nor fibrinolytic therapy was initiated because the criteria for ST-elevation MI were not met [>2 mm ST elevation in two contiguous precordial leads (V1–6), or 1 mm in limb leads (I, II, III)]. These criteria are based on normal population values of up to 2 mm of ST elevation in precordial leads (ACC/AHA guidelines [97]) or ≥2.5 mm in men <40 years of age, or in women up to 1.5 mm (ESC guidelines [98,99]). Serial ECGs on the patient 15, 30, 45, 60 and 120 min from presentation showed borderline changes of up to 2 mm in a single precordial lead (movie), still not reaching the criteria for reperfusion therapy. Urgent angiography was performed, which showed an occluded mid left anterior descending artery. A subsequent echocardiogram showed normal wall thickness and preserved ejection fraction. In retrospect, A-ECG demonstrated serially progressive abnormalities in its derived vectorcardiographic component, horizontal plane (movie), and thus, strongly indicated the presence of an evolving (worsening) acute anterior MI prior to the return of the second troponin measurement, collected 12 h later. All logistic score-related and discriminant analysis-related A-ECG results were also highly positive for acute coronary syndrome (ACS) in all six recordings. The latter results are shown in (Figure 10B).

## Case 2: Chemotherapy Associated Cardiotoxicity

**Figure 11:**
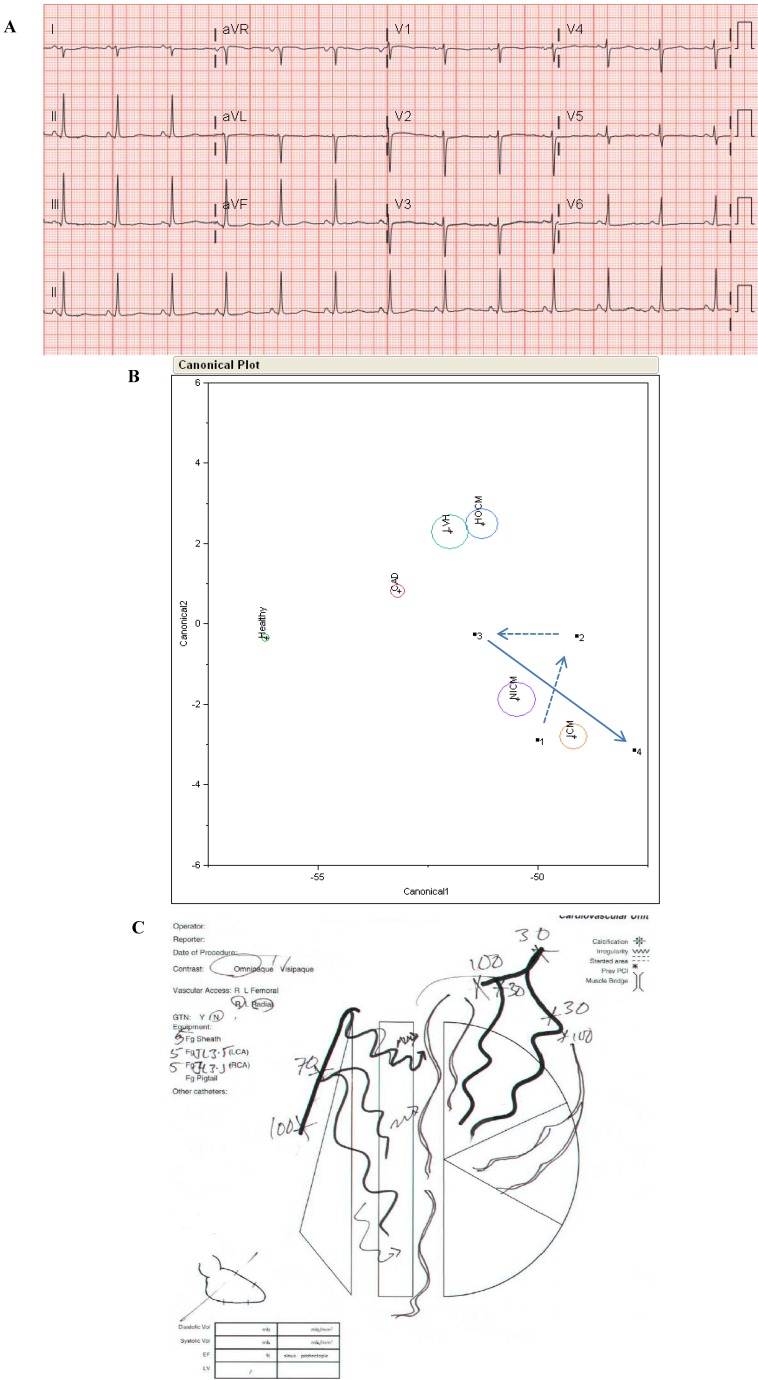
Conventional ECG for Case 2 (**A**); (**B**) A-ECG-related discriminant analysis results for Case 2 using multiple advanced ECG parameters. Red circle = Coronary Artery Disease (CAD) population; Aqua circle = Left Ventricular Hypertrophy (LVH) population; Blue circle = Hypertrophic Cardiomyopathy (HOCM) population; Purple and Orange circles = Non-Ischemic (NICM) and Ischemic (ICM) Cardiomyopathy populations; (**C**) Angiogram results for Case 2: occluded left anterior descending, left circumflex and right coronary arteries.

A 61 year old Maori woman with oestrogen and progesterone receptor positive, HER2 negative breast cancer was treated with an anthracycline based chemotherapy regimen. She presented seven months later with symptoms of heart failure and a clinical diagnosis of anthracycline cardiomyopathy but with unremarkable conventional ECG (Figure 11A). Several months subsequent to that, after initially responding to heart failure therapies, exertional dyspnoea recurred. A cardiac MRI at that time showed a scar pattern consistent with multivessel coronary disease. A-ECG-related discriminant analysis performed blindly in retrospect (Figure 11B) suggested nonischemic and/or ischemic cardiomyopathy at her first presentation (time point 1 in Figure 11B), even prior to the first echocardiogram, then initial improvement during a period of successful medical therapy (time points 2 and 3 in Figure 11B, noting gradual movement of results toward those of a known “healthy population” in the large underlying database), and finally recrudescence (time point 4 in Figure 11B). Results suggest that A-ECG technology was not only capable of making the early non-invasive diagnosis of her condition, but also of non-invasively detecting the initial improvement followed by ultimate deterioration, as well as the definitive eventual diagnosis of ischemic cardiomyopathy (Figure 11C). A coronary angiogram confirmed critical anatomy.

## Case 3: Hereditary Cardiomyopathy

**Figure 12:**
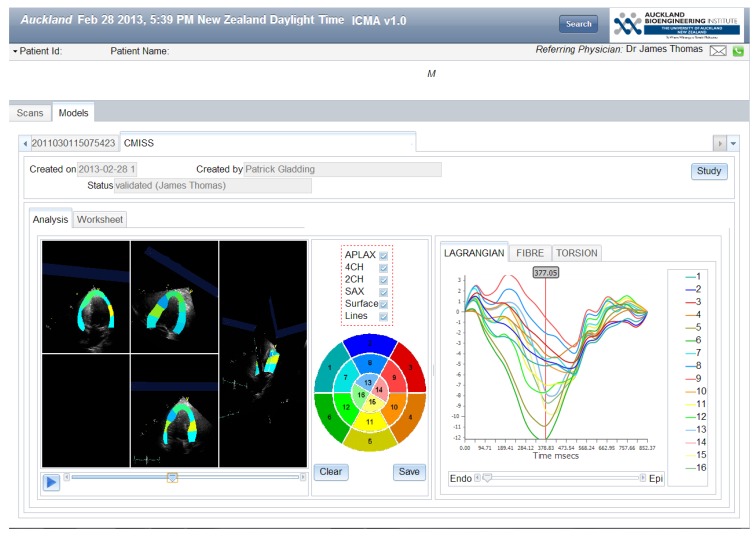
Left ventricular global longitudinal strain (Software engineering by Jagir Hussan).

A 26 year old asymptomatic male athlete with a family history of idiopathic cardiomyopathy, in his father and uncle, presented for screening. Examination and a conventional ECG were considered normal. A transthoracic echocardiogram showed a mildly dilated left ventricle and low-normal ejection fraction 50%–55%. A differential diagnosis of athlete’s heart versus subclinical cardiomyopathy was made. The advanced ECG result was abnormal and global longitudinal strain measured using open access software indicated significantly reduced systolic function GLS −8.7% (Figure 12) (Proprietary software −10.5%; Normal range 18.4% ± 0.4%). Next generation gene sequencing was not available due to the complexity and cost of obtaining this data, despite recent evidence showing Titin gene mutations as an important determinant of hereditary cardiomyopathy [100]. 

## Case 4: Pharmacogenomics and Stent Thrombosis

**Figure 13:**
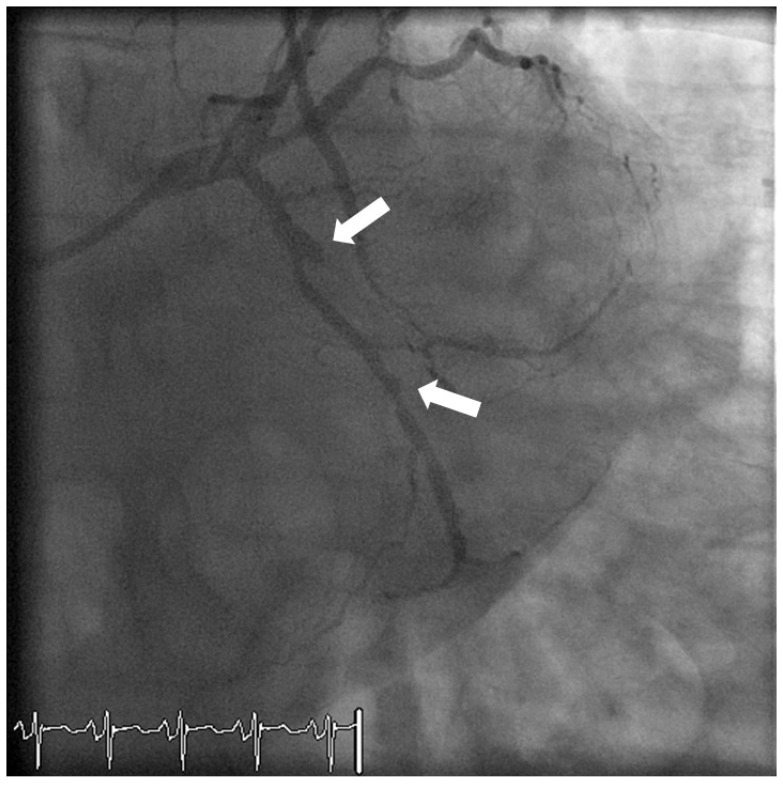
Coronary angiogram demonstrating occluded OM1 and OM2 arteries.

A 41 year old man with a previous history of coronary artery disease presented with TnI negative unstable angina. He underwent an angiogram which proceeded to PCI with a Promus Element™ and second generation Xience™ drug eluting stents to obtuse marginal (OM) one artery and OM2, respectively. The procedure was uncomplicated. He returned five days later with TnI positive chest pain and ST depression on conventional ECG. Both OM stents were occluded with thrombus (Figure 13, white arrows demonstrating occluded branching arteries). An older stent in OM3 remained patent. Rapid genotyping on a Nanosphere Verigene analyser indicated that he was a poor metabolizer of clopidogrel, and a heterozygote for the CYP2C19*2 allele*.* Further stents were deployed to both occluded arteries and he was placed on prasugrel 10mg once daily.

## Case 5: Lamin A Associated Cardiomyopathy

**Figure 14:**
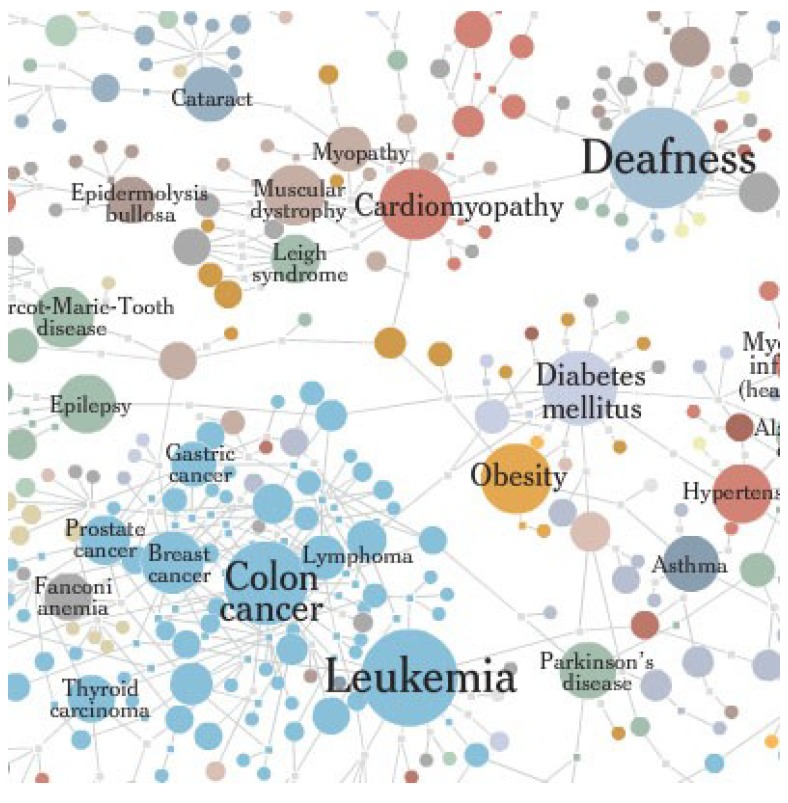
Disease network relationships between cardiomyopathy and muscular dystrophy.

A 31 year old woman with diabetes, lipodystrophy and limb-girdle muscular dystrophy presented with atrial fibrillation and heart failure. An echo demonstrated a severe dilated nonischemic cardiomyopathy. She developed nonsustained ventricular tachycardia and a defibrillating pacemaker was implanted. Heart failure therapy was initiated and she responded but subsequently developed type II respiratory failure. A Lamin A associated disease was suspected (Figure 14 http://diseasome.eu/) and sequencing of the LMNA gene was performed, revealing a missense mutation in Exon 1 c.121C>T which has been previously associated with this condition [101]. A medline search identified treatment options of MEK1/2 inhibitors (currently in Phase I/II for oncology indications) or farnesyltransferase inhibitors. Lack of safety data and availability prevented the use of these agents. A network medicine approach using computational drug repositioning has revealed converging druggable pathways with conventional therapies [102]. Alternative future options for this patient may include siRNA, delivered using nanoparticles, or gene-edited patient-specific induced pluripotent stem cells (iPS) and regenerative medicine [103].
